# Supplementary material for: The causal relationship between the human gut microbiota and pyogenic arthritis: a Mendelian randomization study
Source: Front Cell Infect Microbiol. 2024 Nov 26;14:1452480. doi: 10.3389/fcimb.2024.1452480 (PMC11629706; doi:10.3389/fcimb.2024.1452480)
Supplement: Supplementary file 1 [file DataSheet1.zip › mendelian test/ebi-a-GCST90027732.csv_leave_one_out.pdf]

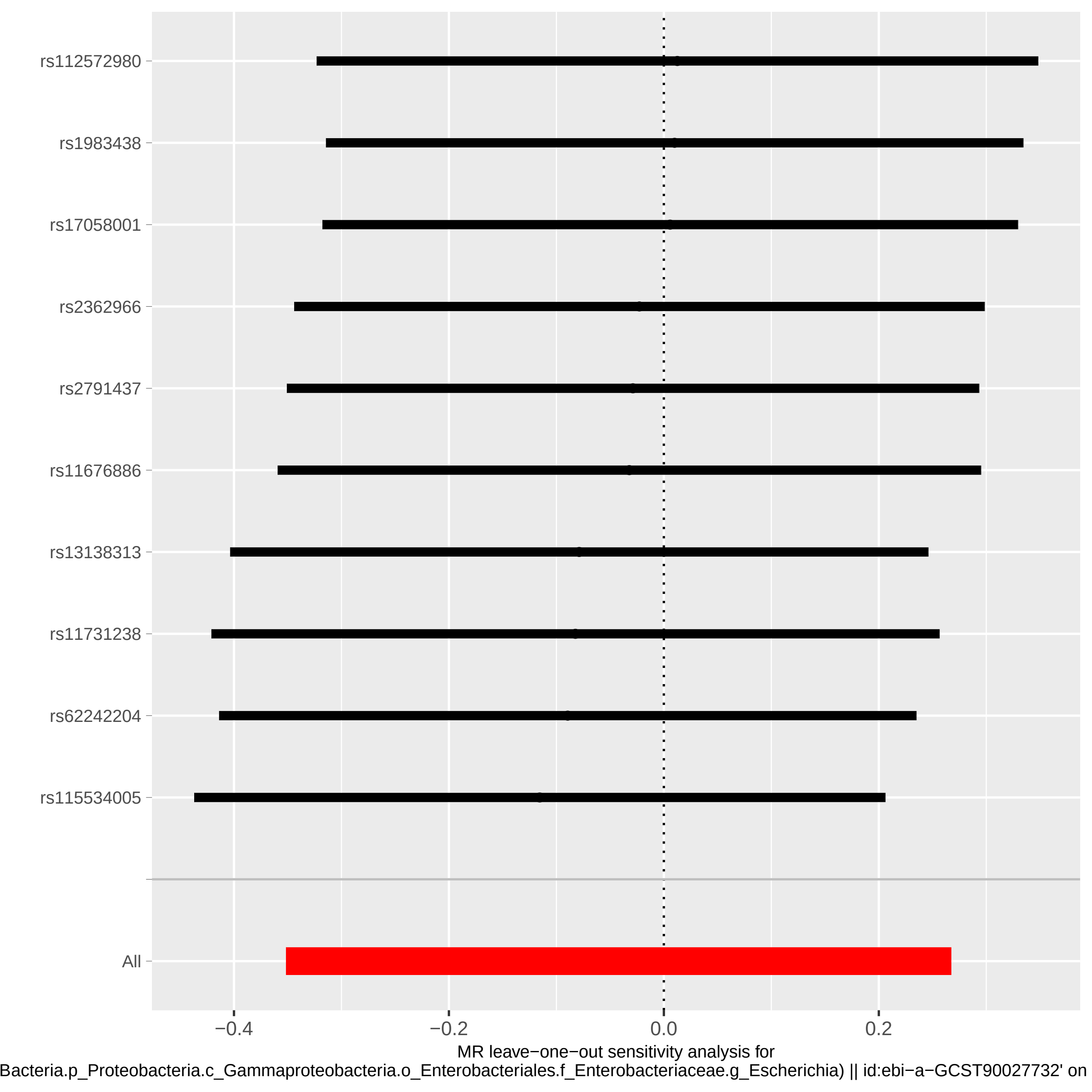
rs112572980
rs1983438
rs17058001
rs2362966
rs2791437
rs11676886
rs13138313
rs11731238
rs62242204
rs115534005
All
−0.4
−0.2
0.0
0.2
MR leave−one−out sensitivity analysis for
Bacteria.p_Proteobacteria.c_Gammaproteobacteria.o_Enterobacteriales.f_Enterobacteriaceae.g_Escherichia) || id:ebi−a−GCST90027732' on
